# Aldolase A deficiency: Report of new cases and literature review

**DOI:** 10.1016/j.ymgmr.2021.100730

**Published:** 2021-02-23

**Authors:** C. Papadopoulos, M. Svingou, K. Kekou, S. Vergnaud, S. Xirou, G. Niotakis, G.K. Papadimas

**Affiliations:** a 1st Department of Neurology, Eginition Hospital, Medical School, National and Kapodistrian University of Athens, Greece; b Laboratory of Medical Genetics, Medical School, National and Kapodistrian University of Athens, "Aghia Sophia" Children's Hospital, Athens, Greece; c Département de Biochimie, Toxicologie et Pharmacologie, CHU de Grenoble, Centre de Référence Rhône-Alpes des Maladies NeuroMusculaires, Grenoble, France; d Pediatric Neurology Clinics, Venizeleion General Hospital, Heraklion, Crete, Greece

**Keywords:** Aldolase A, Rhabdomyolysis, Hemolytic anemia, Aldolase A, ALDOA, glycogen storage disease type, GSD, electromyography, EMG

## Abstract

Aldolase A (ALDOA), is the predominant isoform of aldolase in skeletal muscle and erythrocytes that catalyzes the reversibleconversion of fructose-1,6-bisphosphate to glyceraldehyde 3-phosphate. Autosomal recessive mutations in *ALDOA,* are extremely rare and cause hemolytic anemia and/or recurrent episodes of rhabdomyolysis, usually precipitated by fever. In this report we describe, clinical, laboratory and genetic data of two novel unrelated patients harboring mutations in the *ALDOA* gene who presented with episodic rhabdomyolysis, we review all previously published cases and discuss the most valuable features for diagnosis of this rare disorder.

## Highlights

• Recessive mutations in the ALDOA gene are rare cause of episodic rhabdomyolysis.
• We report two novel, unrelated patients harboring mutations in the *ALDOA* gene presenting with recurrent rhabdomyloysis.
• Patients with ALDOA deficiency show a rather homogeneous phenotype with episodes of rhabdomyolysis, associated either with hemolysis and/or learning disabilities.

## 1 Introduction

Fructose-biphosphate aldolase (Aldolase A, ALDOA), is the predominant isoform of aldolase in skeletal muscle and erythrocytes encoded by the *ALDOA* gene on chromosome 16. ALDOA is a glycolytic enzyme that catalyzes the reversible conversion of fructose-1,6-bisphosphate to glyceraldehyde 3-phosphate [1]. Autosomal recessive mutations in *ALDOA,* are extremely rare and were originally reported to cause hemolytic anemia by impairing thermostability of the enzyme [[2], [3], [4]]. Subsequent reports have identified ALDOA deficiency as a cause of recurrent episodes of rhabdomyolysis, usually precipitated by fever, not always associated with hemolytic anemia, designating the disease as glycogen storage disease type 12 (GSD12) [[5], [6], [7]]. In this report, we describe clinical, laboratory and genetic data of two unrelated novel patients harboring mutations in the *ALDOA* gene who presented with episodes of rhabdomyolysis. We also review all previously GSD12 published cases and discuss the most valuable features for diagnosis of this rare disorder.

## 2 Patients and methods

We present clinical, laboratory and genetic data from two male unrelated Greek patients with *ALDOA* gene pathogenic variants.

### 2.1 Muscle biopsy and biochemical studies

Open quadriceps muscle biopsy was obtained from patient 1 and processed using conventional routine morphological techniques [8]. Aldolase A activity in skeletal muscle was determined as previously described [7].

### 2.2 Molecular studies

DNA was extracted from peripheral blood lymphocytes from patients 1 and 2 and their relatives. Genetic analysis was performed with semi-targeted Exome Sequencing, using Sophia Genetics Clinical Exome Panel (CES) and Nextera Rapid Capture Exome (Illumina), runing on a NextSeq-500 (Illumina USA). The CES panel includes ~4900 genes (114.405 exons), linked to known genetic disorders more than 500 of which are muscle associated Bioinformatic data analysis with SOPHiA DDM (Sophia Genetics) and VarAFT 2.14 was performed. Variants were classified according to the 2015 American College of Medical Genetics and Genomics (ACMG) criteria. In silico analysis of variants included evaluation with tools like PROVEAN, Mutation Taster, SIFT, and Polyphen-2. All results and variant segregation patterns were confirmed by Sanger sequencing when appropriate.

## 3 Results

### 3.1 Clinical findings

Patient 1 is a 24-year-old male of Albanian origin. He was born at term after a normal pregnancy from a non-consanguineous marriage. There was no family history of a neuromuscular disease. His psychomotor development was marked by a language delay which was finally gained at the age of 5 years and growth retardation treated with growth hormone replacement for 8 years. The patient reported that, since the age of 8 years, he presented recurrent episodes of myalgia and dark urine, following long exercise or pyrexia, for which he had never sought medical opinion. Upon presentation, at the age of 23 years, he had mild lordosis and scapula alata, distal hand weakness (interossei graded 3/5 at the MRC scale bilaterally) and neck flexion weakness (3/5 at the MRC scale). Laboratory evaluation revealed high creatine kinase levels (CK, 1500 U/L, normal range 25–190 IU/L), chronic hemolytic anemia and persistently elevated ferritin levels. A forearm lactate test showed normal lactate and ammonia rise. Needle electromyography (EMG) demonstrated small amplitude, short duration, polyphasic motor unit action potentials, with early recruitment both on proximal and on distal upper and lower limb muscles. Nerve conduction studies were normal.

Patient 2 is a 5-year-old boy, born, from a non-consanguineous marriage, at 38 weeks of gestation by caesarian section. On the second day of life, he presented tachypnea and failure to thrive. He was admitted to the neonatal intensive care unit where clinical examination upon admission showed a poor general condition, with a weak cry and a low muscle tone. Laboratory evaluation at the episode, revealed rhabdomyolysis with high CK levels (59,670 U/L, normal range 25–190 IU/L), myoglobinuria, as well as high total bilirubin and ferritin levels. A thorough search for an underlying metabolic disease, including serum carnitine, plasma acylcarnitines, urine amino and organic acids averred negative. The patient was treated with intravenous hydration with subsequent improvement and was discharged a week later in a good general condition and a normal clinical examination. At the age of 2.5 years, and during the course of a viral infection with high fever, he presented a second episode of rhabdomyolysis with CK levels reaching 24,000 U/L, myoglobinuria, high total bilirubin and ferritin levels. He was treated with hydration and was discharged 5 days later. Currently, on last examination, the patient has normal psychomotor development and presents no signs of neuromuscular disorder. His CK levels are moderately elevated (600, U/L, normal range 25–190 IU/L), he has no sign of chronic haemolytic anemia or hypeferritinemia.

The characteristics of all reported cases of *ALDOA* gene mutations associated with rhabdomyolysis and/or myopathy are summarized in Table 1.

**Table 1:** Patients' characteristics.

Patient	Ancestry	Age at first episode (yrs)	Rhabdomyolysis trigger	CPK at rest (U/L)	Hemolytic anemia	EMG	Muscle biopsy	Other	Aldolase A activity in muscle (nmol/h/mg)**	Mutation***	Reference
**P1**	Albanian	8	Exercise, fever	1500	Yes	Myopathic*	Rare atrophic muscle fibers, rare regenerative muscle fibers, increased central nuclei	Growth retardation. Language delay	125	Homozygous c.839C > T (p.Ala280Val)	This report
**P2**	Greek (Crete)	Newborn	Fever	600	During episode	N.P.^$^	N.P.	–	N.P.	Homozygous c.1016G > A (p.Cys339Tyr)	This report
**L1**	German	4 ½	Fever	Normal	During episode	Normal	Variability of both type 1 and type 2 fibers diameter. Some fibers with intracytoplasmic fiber splitting with increased activity of acid phosphatase	Diminished muscle mass, proximal muscle weakness, slightly delayed motor development and language acquisition	9.8^$$^	Homozygous c.619G > A (p.Glu206Lys)	Kreuder et al. [5]
**L2**	Sicilian	Newborn	Fever	13,800	Yes	N.P.	Atrophic and hypertrophic muscle fibers with increased internal nuclei (post mortem)	Proximal muscle weakness. Deceased at 54 months of age due to acute rhabdomyolysis during a febrile illness	N.P.	c.1016G > A, (p.Cys339Tyr)/ c.910C > T, (p.Arg304*)	Yao et al. [6]
**L3,L4,L5**	Moroccan	2 months	Fever	Normal to elevated (up to 1800 U/L)	No	Normal	Excessive lipid droplet accumulation visualized with oil-red-O staining	Learning disability in 2 patients	55	Homozygous c.839C > T, (p.Ala280Val_	Mamoune et al. [7]

* Small amplitude, short duration, polyphasic motor unit action potentials, with early recruitment; ** normal values: 581–5168 nmol/h/mg; ^$^ N.P.: Not performed;^$$^ U/g ***the nomenclature was adapted according NM_184041.5 transcript.

### 3.2 Muscle biopsy and biochemical studies

**Fig. 1:**
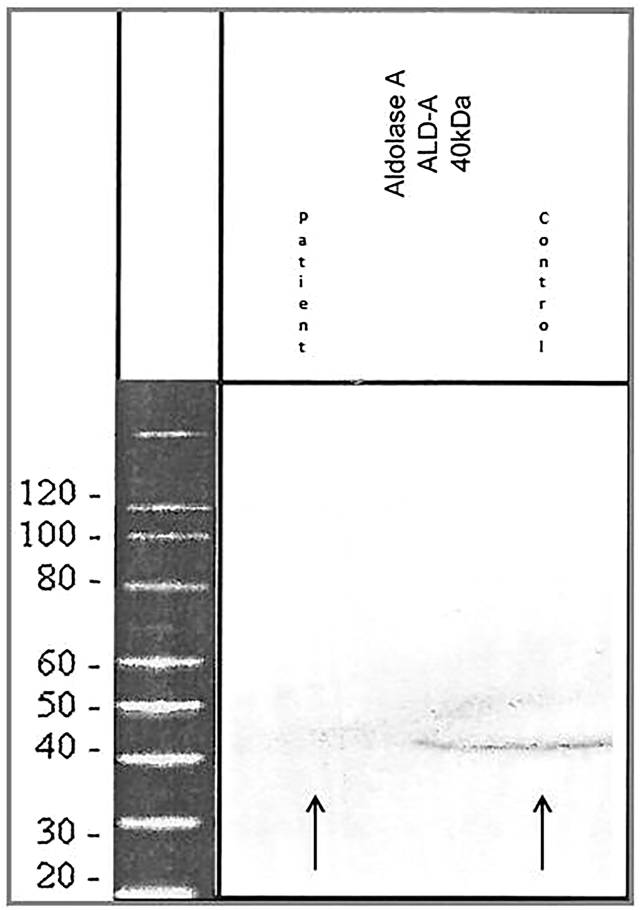
Western blot shows almost complete aldolase A protein levels compared with healthy controls (arrows).

An open deltoid muscle biopsy was performed on patient 1 at the age of 23 years. The biopsy showed non-specific myopathic findings including rare atrophic and regenerative fibers and increased number of central nuclei. ALDOA activity in frozen skeletal muscle was decreased (125 nmol/h/mg; normal 581–5188 nmol/h/mg). Western blot analysis performed on muscle biopsy of patient 1 showed almost complete absence of aldolase A protein compared to control sample (Fig. 1).

### 3.3 Genetic studies

Genetic analysis data identified two known pathogenic variants in the *ALDOA* gene in patients 1 and 2. Patient 1 was homozygous for the c.839C > T (chr16:30081190, NM_184041:exon 8, p.Ala280Val) missense mutation and the status of homozygosity was confirmed by segregation analysis of the variation in the patient's family. Patient 2 carriedthe already reported pathogenic missense mutation c.1016G > A in *ALDOA* gene (chr16:30081454, NM_184041: exon 9, p.Cys339Tyr) in an apparently homozygosity (segregation analysis was not available). Both variants are absent from controls (1000G and gnomAD) and multiple lines of computational evidence such as PROVEAN, Mutation Taster, SIFT, Polyphen-2 support a deleterious effect on the protein.

## 4 Discussion

In this report we describe two novel patients harboring mutations in the *ALDOA* gene. The first patient (P1) presented with episodes of rhabdomyolysis following fever or exercise since childhood and the second patient (P2) episodes of rhabdomyolysis in the context of viral infection since birth. Biochemical studies in patient P1 disclosed reduced ALDOA activity in muscle. Genetic studies showed that patient P1 harbored the previously reported missense c.839C > T (p.Ala280Val) homozygous variation in the *ALDOA* gene [7], while patient P2 was homozygous for the previously reported c.1016G > A (p.Cys339Tyr) missense mutation [6].

The characteristics of all previously reported GSD12 cases are summarized in Table 1. Patients usually present with episodes of rhabdomyolysis starting at the newborn period or early childhood, usually, precipitated by fever. Electromyographic studies are not useful in the diagnosis, since they can be normal or show a non-specific myopathic pattern. Creatine kinase levels range in between attacks from normal to up to very highly elevated and muscle biopsy is not contributing to the diagnosis as it, usually, reveals non-specific alterations. Biochemical studies in muscle, when performed, show diminished ALDOA activity. Most reported patients present hemolytic anemia during episodes or even between attacks, a fact that can narrow down the differential diagnosis, since hemolysis is only associated with aldolase A [5], phosphofructokinase [9] and of phosphoglycerate kinase deficiency [10]. The presence of hemolysis is explained by the low activity of ALDOA in erythrocytes. Patients L3, L4, L5, were reported not to have chronic hemolytic anemia and only presented episodes of rhabdomyolysis [7]. The authors postulated that this was due to greater residual enzyme activity observed in these patients compared to previously reported cases, to the fact that hematological investigations were not performed during episodes of decompensation, or to a selective resistance to thermoliability in erythrocytes [7]. Nevertheless, patient P1 with the same homozygous mutation shows chronic hemolytic anemia not related to acute episodes. Although we didn't measure ALDOA activity in erythrocytes in P1, it seems that the clinical expression of ALDOA deficiency is not exclusively related to mutation type or tissue specific thermoliability and that, probably, other factors contribute to this variable disease expression. These, still unidentified, factors, collectively, could explain the fact that originally, ALDOA deficiency was only reported as a cause of nonspherocytic hemolytic anemia, not related with skeletal muscle symptoms [[2], [3], [4]]. Mental retardation has been reported in the original ALDOA patients [2]. Subsequently reported patients [[5], [6], [7]], as well as patient P1, also showed learning disabilities or delayed language acquisition. Since ALDOA is the aldolase isoform with the most abundant expression in the brain [11], it is not surprising that mutations in the *ALDOA* gene could possibly result in subtle cognitive dysfunction and that brain involvement can be a part of the GSD12 spectrum.

Patient's P1 most usual precipitant of rhabdomyolysis episodes was exercise, unlike previously reported patients, who usually experienced rhabdomyolysis following febrile illness [[5], [6], [7]]. It has been shown that the c.839C > T mutation affects thermostability of the enzyme and that stimulation of patients' myoblasts with TNFa and IL-1β further reduces ALDOA activity [7]. Exercise increases core body temperature [12], while it induces secretion of pro-infalmmatory cytokines, including TNFa and IL-1ß [13]. Probably, high core body temperature and secretion of pro-inflammatory cytokines during exercise, exacerbates ALDOA deficiency leading to exercise-induced rhabdomyolysis [7]. This phenomenon could explain the normal results in the forearm exercise test performed in our patient. During the short duration of a forearm exercise test, performed on ambient conditions, the body core temperature doesn't increase high enough in order to affect the thermostability of the enzyme. It is possible that the residual, decreased, enzymatic activity is sufficient enough for a normal exercise test.

Fixed muscle weakness seems not to be a constant feature of ALDOA deficiency. Some patients show mild proximal weakness at a very young age [5,6], while others, have no signs of muscle weakness until childhood [7]. Upon examination at 23-years of age, patient P1 had distal upper limb weakness, head flexion weakness and showed bilateral scapular winging, while other patients, homozygous for the same, c.839C > T mutation (L3, L4, L5) had no muscle weakness at a younger age [7]. It is unclear if this clinical difference is due to still unidentified additional genes and non-genetic factors such as life-style, nutrition or, yet, unknown environmental modifiers, or if muscle weakness will develop later in the course of their disease.

Patient P2, at the age of 5, shows a rather benign disease course with only 2 episodes of rhabdomyolysis and no signs of muscle weakness at last examination. On the contrary patient L2, heterozygous for the same missense mutation, that modifies the structure of the enzyme in a temperature-sensitive fashion, presented proximal lower limb weakness since the age of 2 and a severe disease course leading to death at 54 months of age. He, additionally, harbored the c.931C > T, nonsense mutation that produces a “null” allele and leads to production of a truncated protein [6]. The presence of a “null” allele led to more severe ALDOA deficiency and decompensation during fever and ultimately to a graver disease phenotype in patient L2, unlike the rest of GSD12 reported patients, who have missense mutations in the *ALDOA* gene*,* interfering with the protein tetrameric structure in a thermoliabile fashion [[5], [6], [7]].

## 5 Conclusion

Patients with ALDOA deficiency show a rather homogeneous phenotype that comprises episodes of rhabdomyolysis, starting at the newborn or early childhood period, precipitated by fever or exercise and associated either with hemolysis or learning disabilities, or both. Although both muscle biopsy and EMG may show non-specific myopathic changes hemolysis and learning disabilities are key features that may aid in achieving the correct diagnosis.

## Funding

This study was supported by grants from the Scientific Society for Rare Disease & Orphan Drugs.

## Declaration of Competing Interest

None.
